# Patient education on Instagram? Structured content analysis of the hashtag “obstructivesleepapnea”

**DOI:** 10.1007/s00405-025-09211-4

**Published:** 2025-01-20

**Authors:** Christopher Seifen, Johannes Pordzik, Katharina Ludwig, Katharina Bahr-Hamm, Christoph Raphael Buhr, Christoph Matthias, Haralampos Gouveris

**Affiliations:** Sleep Medicine Center & Department of Otolaryngology, Head and Neck Surgery, University Medical Center Mainz, Langenbeckstraße 1, 55131 Mainz, Germany

**Keywords:** Social media, Instagram, Obstructive sleep apnea, Content analysis, Patient education

## Abstract

**Purpose:**

Instagram ranks among the most used social media platforms worldwide. An increasing number of posts are dedicated to specific medical topics, such as sleep medicine. The educational content of these posts is largely unknown. Therefore, a structured content analysis of posts linked to the hashtag “obstructivesleepapnea” was conducted, as obstructive sleep apnea (OSA) represents the most common sleep-related breathing disorder.

**Methods:**

The hashtag “obstructivesleepapnea” was entered into Instagram’s search field. The first linked post was selected and then subdivided into visual content and text content for systematic analysis with a focus on educational information on OSA. Demographic factors of the post such as likes, hashtags and the posting account were also included in the analysis. The data collection was completed for *N* = 150 consecutive posts.

**Results:**

37.3% of the visual content and 32.7% of the text content addressed educational information on OSA. In both subgroups, the most frequently discussed aspects were OSA symptoms, comorbidities, and therapy (visual content: 50.0%, 39.3, and 41.1%, respectively; text content: 42.9%, 44.9%, and 24.9%, respectively). The most common (professional) background of the account, as self-stated by the holder, was dentists (29.5%). Additional sleep medicine content was posted by 34.3% of all accounts.

**Conclusion:**

Instagram offers informative content about OSA and is therefore a potential source for patient education. However, the content available is often poorly organized and in most cases incomplete. Patients may have difficulty categorizing the information provided to benefit from it.

## Introduction

Social media is on the rise and has become an integral part of the everyday lives of billions of people. In 2024, the average daily usage time of social media platforms by internet users reached 143 min, 37.5% more than a decade earlier [1, 2]. With more than 1.4 billion monthly active users worldwide, Instagram is now the fourth most used social media platform [3]. Launched in 2010, Instagram was originally a photo-sharing application that allowed users to take and share photos with a corresponding text (called caption) organized by hashtags. As Instagram’s popularity has grown in the last fourteen years, so have its possibilities. Posts are no longer limited to a single image but can contain a series of images and even short videos. In addition, posts can be edited with the help of artificial intelligence or created collaboratively by two users to enhance social interaction.

Besides social interaction and entertainment, Instagram now also plays an important role in various professional fields, including healthcare and medical education. Many healthcare professionals, researchers and medical organizations use the social media platform to share health-related information, exchange research findings and promote public health initiatives [4]. Therefore, social media platforms such as Instagram can function as an effective tool for promoting health literacy as they allow for the visual presentation of complex medical topics in an easily accessible format [5]. And indeed, the number of social media users specifically looking for such easily accessible and cost-free information on medical topics is increasing [6].

In the broad field of sleep medicine, obstructive sleep apnea (OSA) plays a central role being the most common type of sleep-disordered breathing disorder with increasing prevalence in the general adult population around the globe [7, 8]. More precisely, it is estimated that almost one billion people worldwide are affected by OSA, with prevalence in some countries exceeding 50% of the general adult population [9]. From the pathophysiologic point of view, OSA is characterized by neuromuscular uncoupling at the upper airway level resulting in upper airway collapses with concomitant daytime sleepiness and fatigue [10, 11]. The well-known association with serious comorbidities such as arterial hypertension [12], diabetes mellitus [13], coronary artery disease [14], or stroke [15], emphasize OSA as a major public health concern.

In the context of social media, OSA-related content has already been analyzed using the video-sharing platform YouTube and found to be of high quality in some videos, while medical professionals such as otolaryngologists or specific treatment options including sleep surgery were minimally mentioned [16, 17]. However, there is still no study analyzing OSA-related or other sleep medicine-related content on the billionfold used social media platform Instagram. Therefore, we conducted a structured content analysis of Instagram posts that were linked to the hashtag “obstructivesleepapnea”. In addition, we analyzed the extent to which the content on OSA was educational, e.g. for the use of patients.

## Materials and methods

### Systematic content analysis of the hashtag “obstructivesleepapnea”

**Fig. 1:**
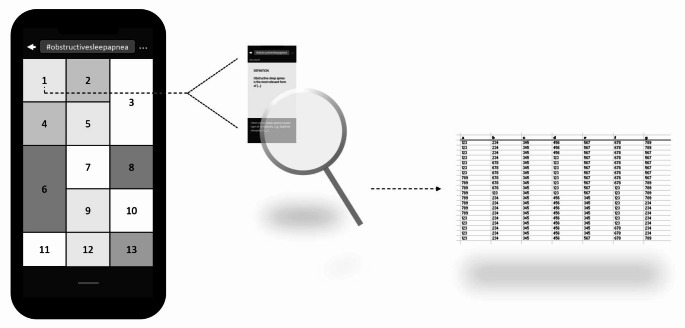
The hashtag “obstructivesleepapnea” was entered in the app’s search field. The linked posts were displayed as shown in this figure. The first post (illustrated as number 1) was selected as the first post for further content analysis

The social media platform Instagram (Meta Platforms Inc., Menlo Park, CA, USA) was used to analyze the content of the hashtag “obstructivesleepapnea”. The content analysis was conducted using the Instagram app version 322.1.0 on an iPhone 12 with iOS version 17.5 (Apple Inc., Cupertino, CA, USA). For data collection, the hashtag “obstructivesleepapnea” was entered in the app’s search field on August 4, 2024, and again on November 4, 2024, using a separate iPhone 12 and Instagram account. According to the app, there were 26,500 and 27,700 posts linked to the hashtag on these two days. To view the linked posts, the hashtag was clicked. Figure 1 shows how the linked posts were displayed in the app.

Of all linked posts, the first was selected and its content analyzed as described below: First, the post was subdivided into its components, visual content (which is required on Instagram) and text content (which is not required on Instagram).

The visual content was classified as an image (single image) or image series (up to ten images). An image series was quantified based on the number of images. The image (series) was then classified into a thematic category based on its content:

- patient education (an actual sleep medicine topic was presented or discussed, e.g. the symptoms of OSA),
- presentation of surgical results (a patient picture was presented in comparison before and after surgery, e.g. for oral surgery),
- advertising (a product was advertised, e.g. a continuous positive airway pressure (CPAP) mask from a mask manufacturer),
- private (a private photograph was shown, e.g. a selfie of wearing a CPAP mask in bed), or
- not classifiable (e.g. an image on which only the word “BIPAP” was written in capital letters without further explanation).

Only if the content of the image (series) concerned patient education about OSA was the content further analyzed (is there information about definition, prevalence, risk factors, pathophysiology, symptoms, diagnosis, comorbidities, or therapy). Finally, the image (series) was checked for a literature source.

The text content was then analyzed. According to the visual content, the text content was classified into a thematic category based on its content (patient education, presentation of surgical results, advertising, private, or not classifiable). Only if the content of the text concerned patient education about OSA was the content further analyzed (is there information about definition, prevalence, risk factors, pathophysiology, symptoms, diagnosis, comorbidities, or therapy). Finally, the text was checked for a literature source.

**Fig. 2:**
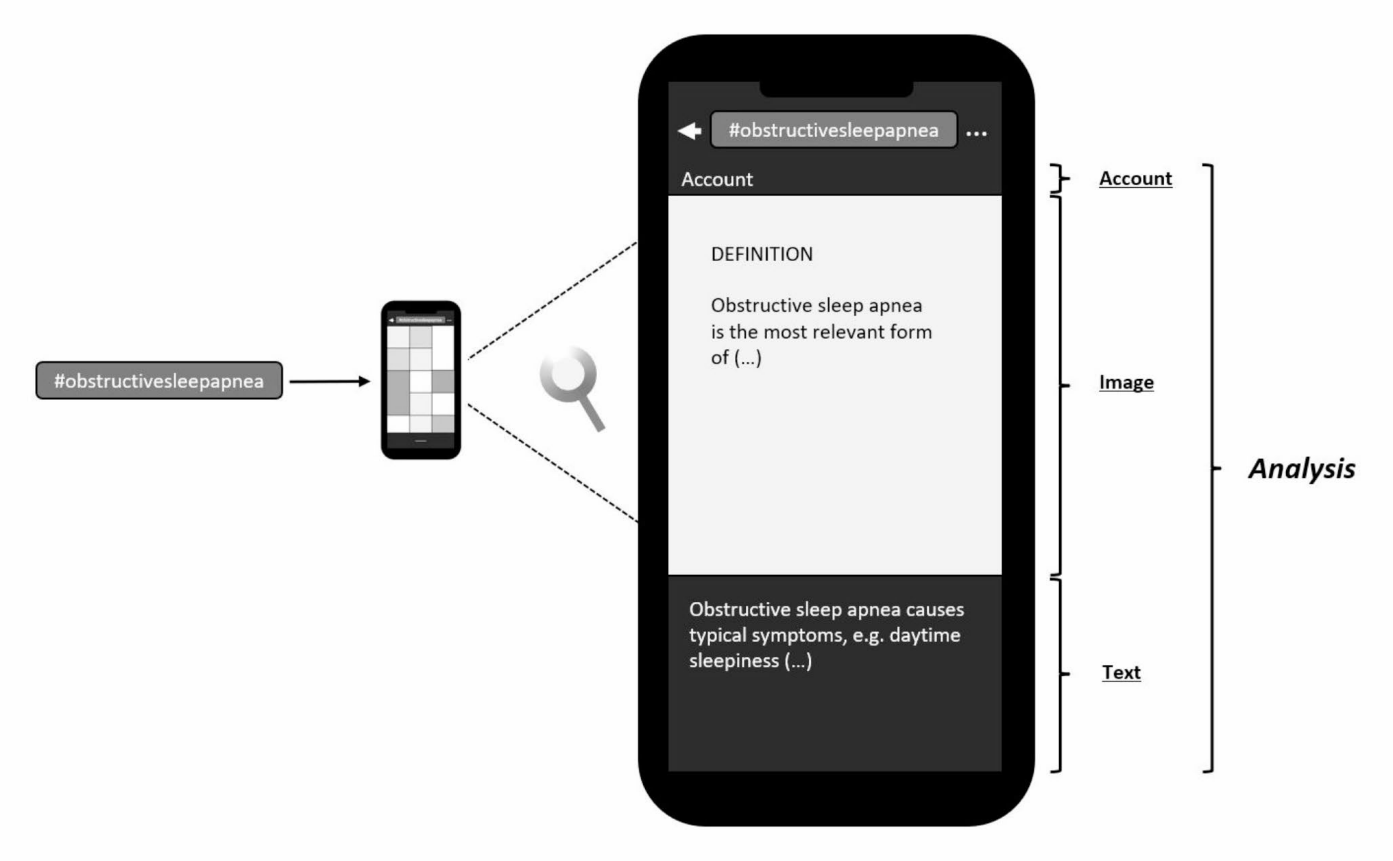
The systematic content analysis was carried out according to previously defined criteria. All posts were subdivided into image content and text content for further investigations. Qualitative and quantitative aspects were both considered

The post was then analyzed for the total number of likes, the total number of linked hashtags in the text of the post, the placement of the hashtag “obstructivesleepapnea” in this sequence and the time of publication of the post. A summary of the systematic content analysis is illustrated in Fig. 2.

After the systematic content analysis of the post, the posting account was further investigated: First, the account was analyzed for the total number of posts, the total number of followers, and the type of account (as specified in the section under the username where information about the account itself is provided). Then the account was checked for verification with a blue mark next to the username. Finally, the account’s last ten posts (images only) were analyzed for content about OSA or sleep medicine in general.

Subsequent to the content analysis of the first post according to the procedure described above, further posts were analyzed by “swiping up”. Through this measure, Instagram would show all existing posts that are linked to the hashtag “obstructivesleepapnea”. However, the data collection was stopped after the content analysis of *N* = 150 posts. To avoid interrupting the app’s automatic algorithm, the data was collected in one piece and without closing the app (*n* = 100 posts on August 4, 2024, and *n* = 50 posts on November 4, 2024). Only posts (image and text) in English were considered.

### Statistics

GraphPad Prism version 5.01 (Boston, MA, USA) was used for statistical analysis. Categorical variables are presented as number and percentage. Continuous variables are presented as mean (M) and standard deviation (SD) when normally distributed vs. median (Md) and interquartile range (IQR) when not normally distributed.

### Ethical statement

No real patient data from our clinic was used for this study. Only content that was publicly available on Instagram has been included in the data collection. The results shown here cannot be traced back to individual posts. All procedures were in full accordance with the Declaration of Helsinki.

## Results

### Visual content analysis

**Fig. 3:**
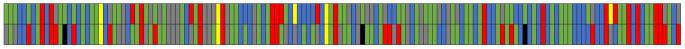
Graphical cluster of all 150 analyzed posts. The upper row shows the thematic classification of the image content while the lower row shows the text content. All content was grouped and color-labeled according to the following thematic categories: Patient education (green),* private (blue)*, *advertising (red)*, *presentation of surgical result (yellow)*, *not classifiable (grey)*, *or posts without text content (black)*

On the days of data collection (August 4, 2024, and November 4, 2024), entering the hashtag “obstructivesleepapnea” in the search field of the social media platform Instagram resulted in 26,500 and 27,700 linked posts, respectively. Of all *N* = 150 posts analyzed, *n* = 55 (36.7%) were single images and *n* = 95 (63.3%) were image series. The image series consisted of Md = 4 (2–7) images. After investigating the main visual content of the post, *n* = 56 (37.3%) were categorized as patient education, while *n* = 5 (3.3%), *n* = 20 (13.3%), *n* = 44 (29.3%), and *n* = 27 (18.0%) were categorized as presentation of surgical results, advertising, private, or not classifiable, respectively. Figure 3 shows a graphical cluster of all 150 analyzed posts.

Further visual content analysis of all posts about patient education on OSA is shown in Table 1.

**Table 1:** Visual content analysis of the 56 educational posts on obstructive sleep apnea

Category, which is addressed in the content	*n*	%
Definition	17	30.4
Prevalence	10	17.9
Risk factors	8	14.3
Pathophysiology	10	17.9
Symptoms	28	50.0
Diagnosis	8	14.3
Comorbidities	22	39.3
Therapy	23	41.1

In *n* = 11 (7.3%) of all visual content, a literature source was cited. All eleven posts in which literature was cited had educational content. This means that 19.6% of all educational image content provided a literature source.

### Text content analysis

There was text content in *n* = 147 (98.0%) of all investigated posts. After investigating the main text content of the post, *n* = 49 (32.7%) were categorized as patient education, while *n* = 3 (2.0%), *n* = 32 (21.3%), *n* = 42 (28.0%), and *n* = 24 (16.0%) were categorized as presentation of surgical results, advertising, private, or not classifiable, respectively (see also Fig. 3). Further text content analysis of all posts about patient education on OSA is shown in Table 2.

**Table 2:** Text content analysis of the 49 educational posts on obstructive sleep apnea

Category, which is addressed in the content	*n*	%
Definition	7	14.3
Prevalence	8	16.3
Risk factors	3	6.1
Pathophysiology	6	12.2
Symptoms	21	42.9
Diagnosis	4	8.2
Comorbidities	22	44.9
Therapy	12	24.3

In *n* = 12 (8.0%) of all text content, a literature source was cited. Ten posts in which literature was cited had educational content. This means that 20.4% of all educational text content provided a literature source.

### Analysis of post characteristics

The evaluation of all posts showed them to have Md = 13.0 (4.0–43.0) likes. The post with the most likes (309 likes) was from category patient education and came from a medical education account. Of all the posts examined, *n* = 64 (42.6%) had less than 10 likes. Of the 25 posts with the most likes (69–309 likes), both the visual and text content of *n* = 13 (52.0%) was from category private and *n* = 8 (32.0%) posts came from private accounts. The number of linked hashtags in the text of the post was Md = 10.5 (6.0–18.0), while the placement of the hashtag “obstructivesleepapnea” in this sequence was at position Md = 5.0 (2.0–11.0). The date on which the posts were created was Md = 10.0 (5.0–19.0) weeks ago.

### Analysis of account characteristics

The evaluation of all posting accounts showed that they had Md = 258.5 (101.3–611.0) posts in total with Md = 898.0 (414.5–2490.0) followers. The evaluation of the account types that was specified in the section under the username is shown in Table 3.

**Table 3:** Self-stated type of account as specified in the section under the username (in alphabetical order). All posts came from 105 different accounts

Account type	*n*	%
Dental academy	1	1.0
Dentist	31	29.5
Hospital	2	1.9
Maxillofacial surgeon	7	6.7
Medical education	6	5.7
Medical equipment	9	8.6
Medical service	1	1.0
Myologist	2	1.9
Ophthalmologist	4	3.8
Otolaryngologist	1	1.0
Otolaryngology clinic	1	1.0
Physician	6	5.7
Physician assistant	1	1.0
Research institute	1	1.0
Private	15	14.3
Psychologist	1	1.0
Sleep clinic	4	3.8
Sleep coach	3	2.9
Sleep physician	3	2.9
Not classifiable	8	7.6

All investigated posts came from *n* = 105 different accounts. The blue mark as a sign for verification was found in *n* = 3 (2.0%) of all accounts. Additional educational content about OSA or related sleep medicine topics were found in *n* = 36 (34.3%) of all accounts.

## Discussion

Studies analyzing content relevant to sleep medicine, e.g. about the condition of OSA, on the social media platform Instagram are still lacking. The aim of this study was therefore to conduct a structured content analysis of Instagram posts linked to the hashtag “obstructivesleepapnea” to assess the extent to which the content was educational, e.g. for patients. We provide evidence that Instagram offers a wide range of content associated with the hashtag “obstructivesleepapnea”, and that most visual and textual content does indeed contain educational information about OSA. However, in most of the posts, the condition of OSA was not fully explained. Symptoms and comorbidities of OSA were most frequently discussed, while risk factors, pathophysiologic explanations, or the diagnostic process were rarely addressed. Very few posts cited a literature source. In addition, a minority of the posting accounts had a blue mark as a sign for verification. Further, a minority of posting accounts provided more sleep medicine-related content. The most common (professional) background of the posting accounts were dentists, followed by other groups of physicians including sleep physicians, otolaryngologists, maxillofacial surgeons and ophthalmologists.

Content analysis of sleep medicine topics as presented on social media platforms is not new. On YouTube, the search term “obstructive sleep apnea” was used to analyze 48 videos, 52.1% of which had educational content, while 65% addressed the first-line treatment of OSA with positive airway pressure [16]. In another study, in which 54 videos on oral appliance therapy for OSA were analyzed, the quality and reliability of the content was found to be highly variable [18]. In addition, a more detailed analysis of 67 videos about hypoglossal nerve stimulation (HNS) as an alternative treatment of OSA revealed an overall poor comprehensibility and quality [19]. Another study analyzed posts about HNS on Facebook and found that mainly queries were reported and discussed, rather than general information about the device or detailed adverse events [20].

A content analysis of Instagram posts from the field of otolaryngology, for example, showed that the patient’s perspective is primarily expressed on the topic of laryngectomy [21]. Another study analyzed laryngology-related content on Instagram and found that a significant portion of the most popular posts were not posted by laryngologists or other medical professionals, raising questions about the reliability of this information [22]. In line with this, an analysis of tonsillectomy on several social media platforms (including Instagram) showed that only 12.5% had educational content, while 63.9% were lifestyle-oriented [23]. Finally, it was shown that in a sample of over 1,000 posts on Instagram on the topic of cochlear implants, most posts were written by patients and only 1.1% by physicians, with a very low overall proportion of educational content [24].

We acknowledge several limitations of the present study: Firstly, only Instagram posts linked to the hashtag “obstructivesleepapnea” were analyzed. It cannot be ruled out that patients affected by OSA use other search terms to obtain health-related information (e.g. “obstructivesleepapnoea”, “sleepapnea”, or “OSA”). Secondly, content on social media platforms is highly dynamic and can change from time to time. The results of a content analysis, as provided by our study, may turn out differently later, as the data curation of the present study was only carried out twice on August 4, 2024, and November 4, 2024. In fact, this limitation is nearly universal in studies analyzing health-related information on social media platforms as their algorithms vary depending on the time of the analysis [25, 26]. However, due to the latency period of three months, we had the opportunity to conduct the content analysis seasonally, once in summer and again in late fall. Thirdly, social media platforms are subject to an algorithm that may display different content depending on the device from which the app is accessed. To address this limitation, two separate iPhones and Instagram accounts were used for the content analysis. However, the app’s algorithm may be shifted depending on which post is selected first. In this study, the first linked post was selected from the unorganized set of all posts, and the content analysis was performed starting from there. This could be similar to, or at worst deviate from, how patients actually obtain medical information on social media. As we did not conduct a random sample analysis, the presented results may be individually biased due to the applied methodological approach. Fourthly (and probably most importantly), the present study did not check the content with educational content for medical accuracy. A statement about the correctness of those posts with educational content can therefore not be made in this study. The question therefore arises as to what extent the educational content on sleep medicine topics offered on Instagram has the required level of detail to provide patients with sufficient information. Fifthly, only Instagram posts in English were included in the present study, so that the neglect of other languages may have resulted in a biased evaluation of the content about OSA, as a condition with global impact. Sixthly, the presented results are based on the content analysis of 150 randomly selected posts. Therefore, it cannot be ruled out that the analysis of more posts could lead to a different result.

Accepting these limitations, this is the first study that categorizes a random sample of the more than 27,700 (as of November 4, 2024) existing posts on Instagram linked to the hashtag “obstructivesleepapnea”. Future studies should include a larger number of posts in their content analyses to verify the presented results and improve their generalizability. In addition, future studies should also test if the app’s algorithm has an influence on the displayed posts, for example by analyzing posts from more than two devices and accounts. Finally, there is a need to check the accuracy of (and possibly edit) medical content on Instagram to reduce the spread of misinformation and to promote patient safety.

## Conclusion

The existing posts on Instagram linked to the hashtag “obstructivesleepapnea” offer a wide range of information on the condition of OSA. However, the educational content is often poorly organized and, in most cases, incomplete. Therefore, it can be difficult for patients to categorize the content and filter it for educational purposes. Although Instagram posts seem promising for patient education in the field of sleep medicine, healthcare professionals need to be encouraged to post scientifically proven information or refer users to trustworthy sources.

Declarations.
